# An end-to-end software solution for the analysis of high-throughput single-cell migration data

**DOI:** 10.1038/srep42383

**Published:** 2017-02-13

**Authors:** Paola Masuzzo, Lynn Huyck, Aleksandra Simiczyjew, Christophe Ampe, Lennart Martens, Marleen Van Troys

**Affiliations:** 1 VIB-UGent Center for Medical Biotechnology, VIB, Ghent, Belgium; 2 Department of Biochemistry, Ghent University, Ghent, Belgium; 3 Department of Cell Pathology, Faculty of Biotechnology, University of Wroclaw, Wroclaw, Poland

## Abstract

The systematic study of single-cell migration requires the availability of software for assisting data inspection, quality control and analysis. This is especially important for high-throughput experiments, where multiple biological conditions are tested in parallel. Although the field of cell migration can count on different computational tools for cell segmentation and tracking, downstream data visualization, parameter extraction and statistical analysis are still left to the user and are currently not possible within a single tool. This article presents a completely new module for the open-source, cross-platform CellMissy software for cell migration data management. This module is the first tool to focus specifically on single-cell migration data downstream of image processing. It allows fast comparison across all tested conditions, providing automated data visualization, assisted data filtering and quality control, extraction of various commonly used cell migration parameters, and non-parametric statistical analysis. Importantly, the module enables parameters computation both at the trajectory- and at the step-level. Moreover, this single-cell analysis module is complemented by a new data import module that accommodates multiwell plate data obtained from high-throughput experiments, and is easily extensible through a plugin architecture. In conclusion, the end-to-end software solution presented here tackles a key bioinformatics challenge in the cell migration field, assisting researchers in their high-throughput data processing.

Cell migration plays a major role in several physiological processes, such as wound healing and neuronal development, but also in disease conditions such as inflammation processes, and cancer cell invasion and metastasis^1^,^2^,^3^. Cell migration thus provides possible therapeutic targets, for example in immune therapy against cancer, stem cell-based therapies, or regenerative medicine^4^,^5^.

Cell migration is controlled by the integration of multiple mechanical and chemical cues across time and (subcellular) space. This impacts the dynamics of the cellular cytoskeleton, and determines a broad range of different cellular morphologies and migration phenotypes^6^,^7^,^8^. Moreover, many of the underlying processes are stochastic, resulting in heterogeneous migration behavior. The systematic study of cell migration therefore requires high-throughput methodologies that enable not only visualization of the cell dynamics but also in depth quantification of cell motile behavior in space and time^9^. A broad range of experimental tools have thus been developed to investigate cell migration^10^,^11^,^12^, and a rich collection of computational algorithms and software has been produced as well^13^.

Cells can either migrate individually (single-cell migration) or can move together in cell sheets or strands (collective cell migration). The latter is classically investigated using a wound-healing assay in which a scratch is created in a confluent cell layer, and quantification is achieved by measuring changes of wound size in time and deducing migration rates. Although several software tools are available to either quantify the wound area, or the cell-covered area^12^,^13^,^14^, no data management solutions existed for these high-throughput experiments. We therefore previously developed CellMissy, which is the first tool to integrate the management, processing and analysis of collective cell migration data^15^. However, when investigating single-cell migration, the individual motile behavior of cells and its quantification is the focus of the analysis, which uses the coordinates of individual cells over time generated by cell tracking.

Table 1 shows an overview of some of currently available software tools for the visualization and validation of high-throughput, time-lapse microscopy image data of individually migrating cells or objects^16^,^17^,^18^. Despite this table not being comprehensive, it clearly indicates that the field is still lacking a user-friendly, freely-available and open-source software that provides an end-to-end solution for automated management, processing and inspection of the data generated by the tracking software. Such a tool needs to allow for quality control, cell migration parameter extraction, and statistical comparison of different conditions. Proprietary software solutions exist (such as Metamorph, Imaris or Volocity) that provide tools to perform (manual) editing of cell trajectories, that calculate object and track statistics, and that can export selected data. These commercial tools however, are bound to the vendor’s imaging systems. The plethora of free, mostly open-source, solutions for single-cell segmentation and tracking are instead focused on solving the tracking problem only, leaving downstream analysis to the user’s choice^16^,^19^.

The absence of an open solution for automated data management, inspection, quality control, and analysis constitutes the major bottleneck in the processing of high-throughput single-cell migration experiments today, because this process remains very labor-intensive (*e.g.*, cell trajectories are manually inspected and/or data necessarily have to be transferred between different software tools). As a consequence, these data are frequently insufficiently mined^20^. Cell speed and persistence of motion for instance, are only calculated at the cell population level, thus averaging those parameters across a collection of recorded cells, *de facto* losing a lot of potentially interesting information. To fill this gap, we here present an open-source solution, implemented as new module in CellMissy, for the automated quality control, inspection, and analysis of single-cell migration experiments.

## Results and Discussion

**Figure 1:**
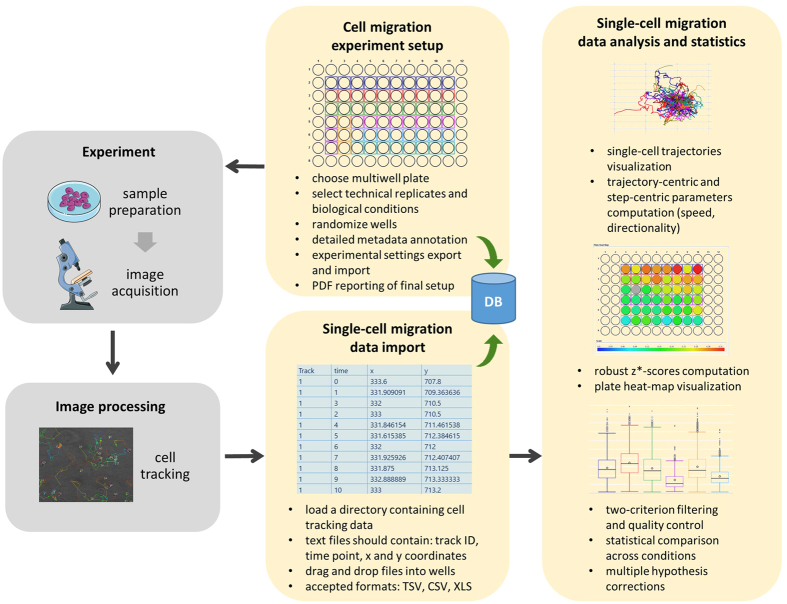
Schematic workflow of the single-cell analysis module presented in this article. Yellow boxes highlight the steps in which the modules operates, together with the broader CellMissy framework and database. The key functionalities of the software are listed for each block. Images in the ‘Experiment’ block by Servier Medical Art, distributed under a CC-BY 3.0 license (http://www.servier.com/Powerpoint-image-bank, https://creativecommons.org/licenses/by/3.0/).

This article presents a free, cross-platform and open-source solution for the automated inspection, quality control assessment and analysis of high-throughput single-cell migration experiments (*e.g.*, in a 96-well format). The platform, developed as a new module in the CellMissy software, and illustrated in Fig. 1, takes text files containing 2D single-cell migration data from a time-lapse experiment as input. These text files take the form of spatial (x, y) coordinates in time, and can be extracted from the user’s choice of cell tracking software. These data can be derived from different experimental setups. Typically, cells are sparsely seeded either on a surface or on a layer of extracellular matrix and then imaged in time. The cells can be assayed after applying a chemotactic gradient (chemotaxis) or not (random migration). Recently, microfluidic systems for chemotaxis assays have become increasingly available^21^. Alternatively, cells can be seeded as a confluent layer in part of the well (*e.g.*, in the well periphery in a cell-zone exclusion assay, or in the center in a fence assay (for more details on assays see ref. 11). Single-cell migration data can also be recorded by imaging in time the single cells escaping from multicellular spheroids or tumor organoids^22^.

**Figure 2:**
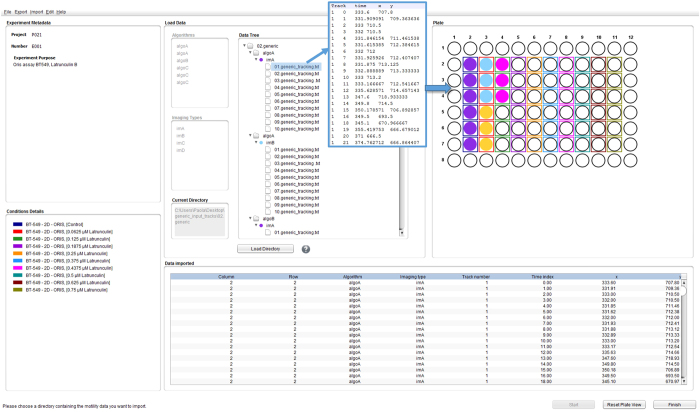
A typical multi-sample single-cell migration experiment loaded in CellMissy. A breast cancer cell line (BT549) was left untreated (control) or treated with a concentration range of a drug (*in casu* nine different concentrations of the actin drug latrunculin B). Each condition on the plate is color coded (see list bottom left and colored boxes around wells in the plate view). Data produced by image processing from the replicate wells are loaded (filled color coded wells in plate view and central list). Each replicate data set (from one well) is a collection of single-cell trajectories, as shown in the text file in the inset. The table at the bottom reports the same data after loading. It is possible to load data associated with multiple image processing algorithms (*Algo*, see central part of view) and/or multiple imaging types (*Im*).

Data are typically derived from a common multiwell plate experimental setup, as shown in Fig. 2, in which multiple conditions are tested in parallel, *e.g.*, with different cell lines and/or different treatments, such as sets of chemical compounds, concentration ranges of a specific compound, selected libraries of siRNA and so on. Multiple technical replicates (*i.e.*, multiple wells) are usually performed for each condition, and the module allows data visualization and analysis at both the condition and replicate level. Obviously, less complicated experiments can also be imported and analyzed. As shown in Figs 1 and 2, the module expects text files reporting four distinct columns: (i) the trajectory unique ID, (ii) the time point, (iii) the x coordinate (either in pixel or μm), and (iv) the y coordinate (either in pixel or μm). This is described in detail in a dedicated user manual at the project homepage.

**Figure 3:**
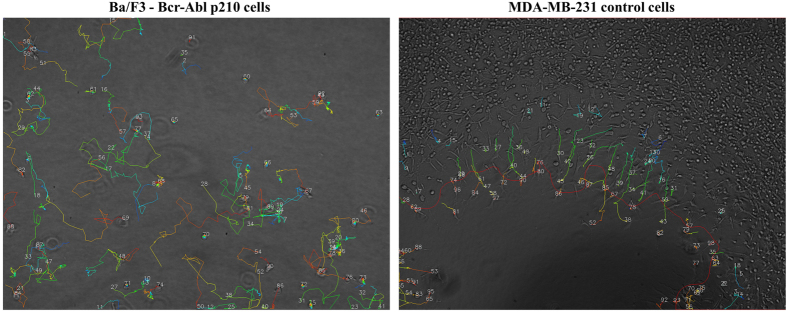
Single-cells trajectories acquired by time-lapse imaging and image processing. Cell trajectories (colored lines) obtained from applying tracking software on a time-lapse image sequence and overlaid on the last image of the sequence for Ba/F3 cells expressing p210 Bcr-Abl (left, experiment 1, sparsely seeded cells, Table 2) and for untreated MDA-MB-231 cells (right, experiment 2, cell exclusion zone assay, Table 2). Numbers indicate individual cell trajectories. Colors indicate temporal evolution of x and y cell positions (blue: earlier in time, red: later in time). The red line in the right image indicates the border between the confluent cell layer and the cell-free zone that has shifted in time. Single cells are only identified as such in experiment 2 when they escape the expanding cell sheet.

Once the data associated with an experiment are loaded, the software provides the following four key functions: (i) data inspection and visualization, (ii) parameter extraction, (iii) quality control, and (iv) statistical data analysis. The following sections describe these functionalities in detail using two multi-sample data sets as examples. These data sets were selected because they were generated *via* different assay types (Fig. 3), using different cell types (immune cells *versus* cancer cells). Furthermore, these data sets reflect a different level of complexity (*i.e.*, number of samples tested in parallel). The details for each experiment are reported in Table 2. Single-cell trajectories from a single replicate in each of the experiments are visualized in Fig. 3. The experiments were performed in phase-contrast, but other imaging techniques are of course also fully supported. The complete single-cell migration analyses for these experiments are available on figshare (see “Methods” section for details).

### Data inspection and visualization

**Figure 4:**
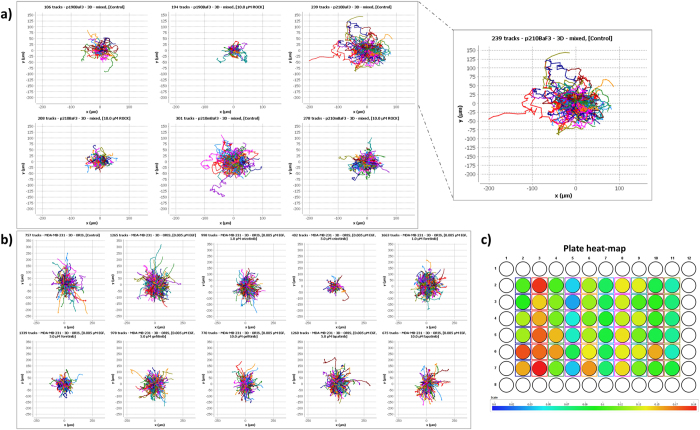
Visualizing the effects on migration via rose plots of single-cell trajectories. Each rose plot visualizes all cell trajectories present in the tested biological conditions (cumulated data of the replicates per condition, number of trajectories are indicated on the top left of each panel). The origin of all trajectories has been shifted to (0,0). (**a)** Six conditions of experiment 1; the rose plots for conditions 2, 4 and 6 where cells were incubated with ROCK inhibitor clearly demonstrate the inhibitory effect. Zoomed view of condition 3 is shown in the box. (**b)** Ten conditions of experiment 2; condition 1: no treatment, condition 2: EGF treatment, conditions 3–10: EGF plus various drugs. Note *e.g.*, the drug concentration-dependent effect in condition 3 *vs.* 4, and 5 *vs.* 6, treated with 1 and 5 μM of crizotinib or foretinib, respectively. (**c**) Plate heat-map visualization of experiment 2: each well in the plate color encodes median cell speed.

Upon starting the single-cell analysis module, the user is presented with the “Data Inspection and Cell Tracks” view. Here, single-cell trajectories imported in CellMissy can be visualized for each replicate (*i.e.*, each well), or across all replicates for a specific biological condition. These plots can either use all trajectories, or only a random subset (with a user-defined sample size) in case of very large data sets. The visualization can either be based on the raw cell trajectories, or on re-centered trajectories where the starting point of each track is located at the origin of the coordinate system (usually depicted in the literature as a ‘rose plot’). It should be noted that the visualization of rose plots for all the biological conditions of an experiment, with identical axis scaling as provided by the module, provides a fast yet powerful way to visually compare migration behavior in diverse settings. This enables the detection of possible differences in cell migration phenotypes, *e.g.*, upon drug treatment in the experiments used here, or differences in response to a gradient in chemotaxis experiments. Figure 4 shows this kind of visualization for the two experiments described in Table 2. Note, for example, that the three Ba/F3 BcrAbl cell conditions treated with Rock-inhibitor y27632 cover shorter distances (Fig. 4a). Furthermore, the multiwell plate of the experiment under analysis can be visualized as a heat-map where each well in the plate color encodes one of the four parameters: (i) number of cell trajectories, (ii) mean or median cell speed, (iii) mean or median cell directionality, and (iv) robust z*-score^23^. An example of this visualization is shown in Fig. 4c.

### Trajectory-centric and step-centric parameter extraction

**Figure 5:**
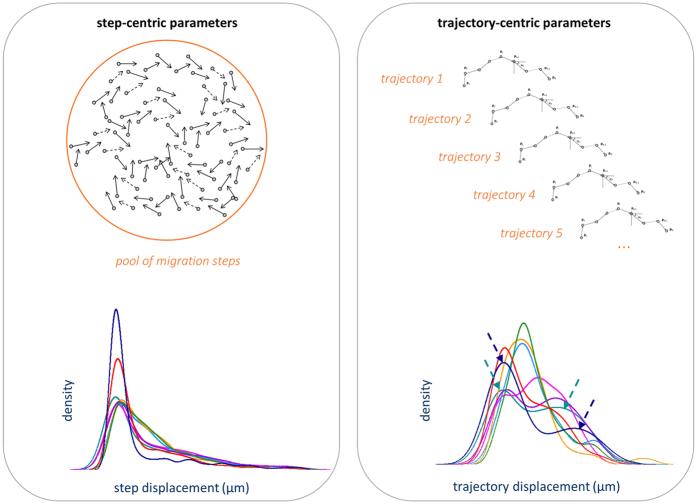
‘Step-centric’ and ‘trajectory-centric’ cell migration parameter computation: complementary analysis approaches. Top panels: schematic representation of the two approaches. Bottom panel: kernel density estimation (KDE) plots of step-centric and trajectory-centric cell displacements *d*_*i*_: the possible presence of cell subpopulations (shown by arrows for two distributions in lower right panel) is overlooked in the step-centric approach. Data are from the experiment described in Fig. 2 (breast cancer cell lines treated with nine different concentrations of an actin drug). The density plots estimate the probability density function of a random variable, *i.e.*, the relative likelihood for the variable to take on a given value. The probability of the variable to fall within a particular range of values is given by the integral of the variable’s density over that range, corresponding to the area under the curve.

**Figure 6:**
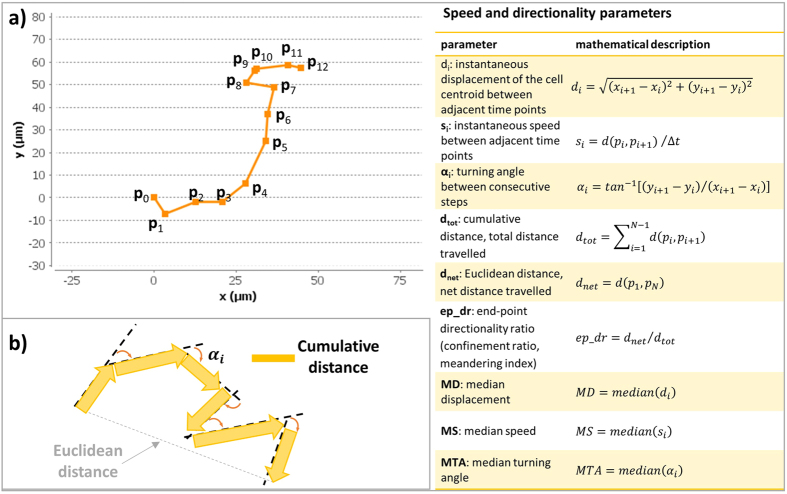
Parameters extracted from a single-cell trajectory in the 2D Euclidean space. (**a**) Schematic representation of a cell track consisting of 12 steps. The parameters reported in the table on the right are computed for each trajectory. (**b)** Schematic representation of net and cumulative Euclidean distance travelled by the cell. The turning angles *α*_*i*_ between successive time frames are shown.

There are generally two different ways to compute cell migration parameters and therefore conduct data analysis, as schematically shown in Fig. 5. On the one hand, one can first carry out a calculation of a specific parameter for each tracked cell separately (we here refer to these as ‘trajectory-centric’ parameters), and then compute an aggregated value from these distributions (mean or median) (see Fig. 5, right). One migration parameter is the instantaneous displacement *d*_*i*_ (defined in Fig. 6a). The distribution of the ‘trajectory-centric *d*_*i*_’ or ‘track *d*_*i*_’ of all trajectories in a cell population (plotted as probability density plot in Fig. 5, bottom right) contains these trajectory-centric values, calculated by *d*_*tot*_/*N*, where *d*_*tot*_ is the total displacement per trajectory and *N* is the number of steps in this trajectory. On the other hand, a distribution and an aggregated value (mean or median) of all separate migration steps in a data set can be calculated, independent of which cell trajectory migration steps belongs to (we here refer to these as ‘step-centric’ parameters) (see Fig. 5, left). Both these quantification methods have their advantages and disadvantages and can therefore complement each other. Consequently, it is useful to combine these in a complete cell migration data analysis pipeline.

A trajectory-centric approach is in general preferable, because this is more intuitive. In addition, this approach is required to address the heterogeneity in cell migration behavior across a cell population. Indeed, the cell population under analysis may consist of different sub-populations of cells that show different and biologically relevant migratory patterns. Such differences will be discovered by plotting the distribution of extracted migration parameters on a trajectory-centric basis, while in a step-centric approach these subpopulations will be overlooked. This is demonstrated for displacement *d*_*i*_ in the bottom panels of Fig. 5. The density plot of the trajectory-centric *d*_*i*_ reveals that in this data set two or more distinct cell populations are present in several replicates of the condition (see Fig. 5, bottom panels, potential subpopulations indicated with dashed arrows). This aspect finds an important application in drug design, where targeting of specific populations in a complex cell mixture (*e.g.*, containing tumor and stromal cells) is sometimes desired^24^,^25^.

On the other hand, trajectory-centric parameters can be biased for several reasons^26^. First of all, the number of steps that are monitored can vary a lot between cells, and this can affect the calculated parameters. Second, if a cell population is imaged with a certain time interval, fast moving cells tend to be associated with relatively straight trajectories, high confinement ratios and low turning angles. Therefore, they could be seen as a distinct subpopulation. Researchers frequently also discard cell trajectories shorter than a certain time period from their analysis, thus possibly introducing another bias towards more slowly translocating cells.

Another source for bias in trajectory-centric parameters comes from artefacts intrinsic to the tracking algorithms. For example, both the splitting of cell trajectories as well as the re-entry of cells in the field-of-view can cause a single cell to contribute more than once to a trajectory-centric parameter. This problem is overcome in a step-centric analysis, because it does not matter to which cell a movement step belongs. Even the switching of trajectories does not constitute a major problem in this case, because only the steps involved in the switch are affected rather than the entire trajectory (as would happen in trajectory-centric analysis).

**Figure 7:**
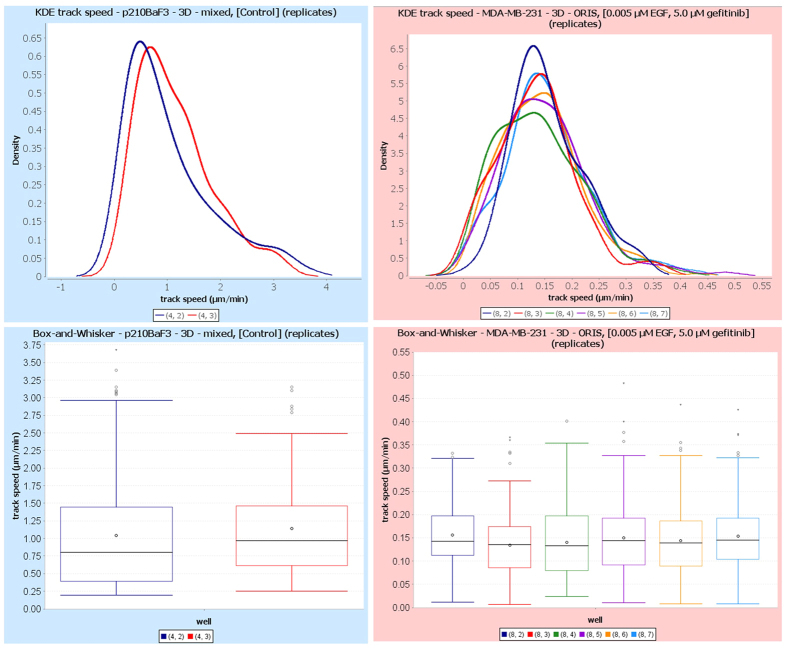
Cell speed distributions are visualized via kernel density estimation (KDE) plots and boxplots. Speed distributions are shown for the two replicates of condition 3, experiment 1 (untreated p210Bcr-Abl Ba/F3 cells, left), and for the six replicates of condition 7, experiment 2 (0.005 μM EGF + 5.0 μM gefitinib, right). Different colors correspond to different technical replicates or wells, as shown in legends. Note the difference in distribution range for the two cell types, with the immune cells (experiment 1, left) displaying faster migration. The box plots show the median (line) and the mean (point); boxes indicate interquartile ranges (Q1–Q3), while whiskers span the minimum and maximum of the data. Dots represent outliers, and triangles far outliers.

**Figure 8:**
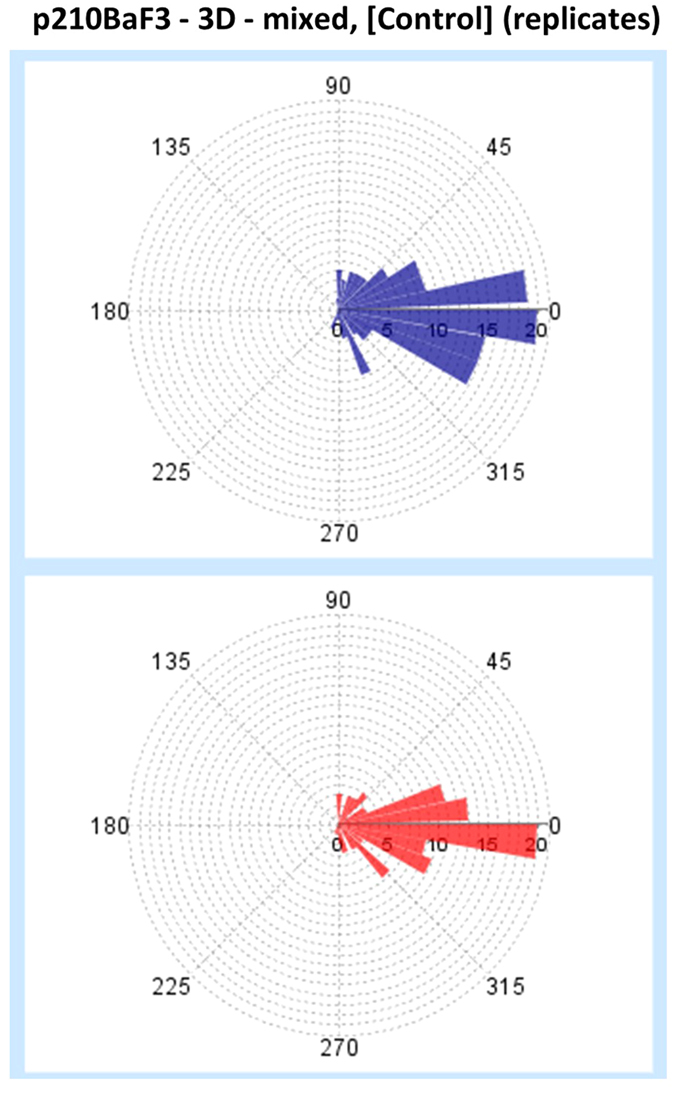
Cell turning angle distributions are visualized via angular histograms. Turning angle distributions are shown for the untreated p210Bcr-Abl Ba/F3 cells (condition 3, experiment 1). Each distribution corresponds to a specific technical replicate. The angular bin is 10 degrees.

For these reasons, we have chosen to present a framework that allows for both these computations to take place at the same time, enabling researchers to use a step-centric or a trajectory-centric approach, or even a combination of these approaches. Figure 6 illustrates the quantitative parameters that are currently derived in our new single-cell analysis module. Given the (x, y) coordinates in time, each cell trajectory is described as a collection of points in the 2D Euclidean space, as shown in Fig. 6a. From these coordinates, displacement *d*_*i*_ and (track) speed *s*_*i*_ (in pixel or μm and pixel/min or μm/min, respectively) are computed as listed in Fig. 6. The distributions of the step-centric and trajectory-centric displacements, and of the cell speed for each condition at the replicate level are reported using boxplots and kernel density estimations. The latter are particularly useful for differentiating between unimodal and multimodal distributions. These two types of plots are illustrated for track speed in Fig. 7. Cell turning angles *α*_*i*_ between successive time frames (see Fig. 6b) are computed (both step-centric and trajectory-centric) as a speed-independent indicator for directionality, and are presented through traditional as well as angular histograms (see Fig. 8). Furthermore, *ep_dr*, end-point directionality ratios (also known as confinement ratios or meandering indices) are computed trajectory-centrically as ratios between the net distance and the cumulative path traveled by the cell (see Fig. 6).

All of the visualizations discussed on this section can be made on both the replicate level as well as the condition level.

### Data quality control: applying a minimal-motility threshold using a two-step filtering approach

Artifacts are always present in data sets generated at high throughput, due to variation in positioning during automated image acquisition, errors during segmentation, and errors in defining the center of mass of segmented objects during automated tracking. As a consequence, non-moving objects (dead cells, non-cell particles, artifacts segmented due to differences in contrast, etc.) are assigned a non-zero displacement (or speed) by the image processing software. Finding and applying a lower limit or threshold on these variables is thus desired when cell tracking has been performed fully automatically. Obviously, this filtering should not remove relevant data and therefore needs to be flexible.

In the single-cell analysis module, the raw data are run through a quality control step, which is implemented with a minimal-motility filtering: only trajectories that meet a tunable two-step filter for minimal cell motility are retained for final analysis.

The first criterion in this two-step filter is step-centric: cell steps are either labelled as ‘true’ or ‘false’ if these are larger or smaller than a minimal translocation, respectively. This minimal translocation can be specified (in pixels or μm) by the user. The second filter criterion is trajectory-centric: trajectories are only retained if they meet the first criterion in at least a specified percentage of their steps (*e.g.*, 30% motile steps/trajectory). Based on our experience with different cell lines and inhibitors, a minimal translocation/step of 0.1–0.5 μm (for immune cells) and 1.6–2 μm (for cancer cells), and between 20 and 30% of motile steps/trajectory appear suitable defaults for these two criteria. Of course, these criteria may vary depending on the effect of a treatment, *e.g.*, when using a strong inhibitor of cell migration.

**Figure 9:**
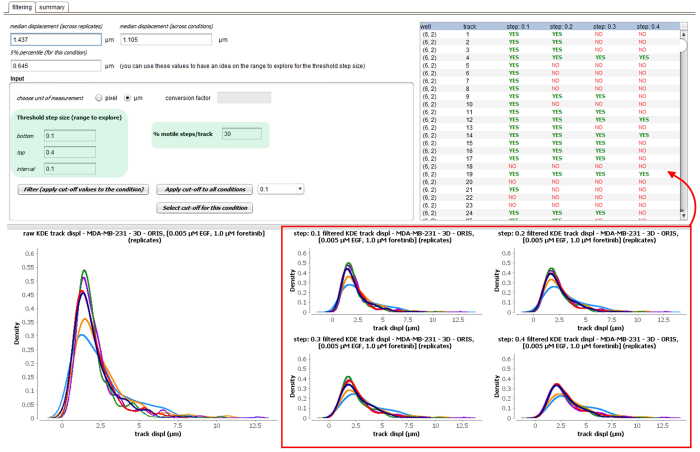
The quality control view of the software: two-step thresholding. The user can input a range for the threshold step size (*i.e.*, displacement) to be tested (box on the left). The choice is guided by displaying the median and 5% percentile of the displacement distribution for the condition (across replicates) and the experiment (across conditions). Second, the user inputs a percentage of desired motile steps per cell trajectory (box on the right). The effect of each tested threshold value for the displacement can be evaluated through the density plots of the track displacement distributions (red box). For each tested value (columns), the table reports the list of cell trajectories (rows) marked in green (YES) if retained, and in red (NO) if not retained. This functionality is here shown for condition 3 of experiment 2.

**Figure 10:**
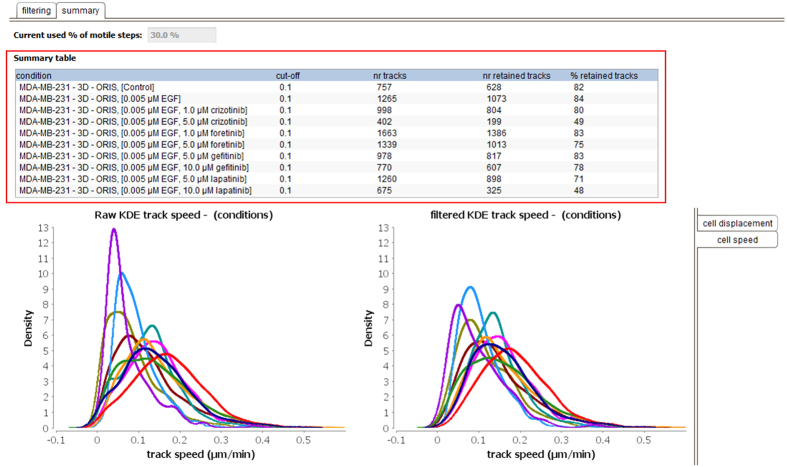
The summary filtering view of the software. The summary table (red box) reports the cut-off used for each condition of the experiment, together with the initial and the retained number of cell trajectories. The final effect of the filtering can be evaluated with the density plots of raw and filtered displacement and speed distributions (bottom panels, different colors represent biological conditions reported in the table). This functionality is here shown for experiment 2; control in dark blue, high dose crizotinib in purple, and high dose lapatinib in olive green.

The module therefore allows a range of criterion values to be tested simultaneously. Evaluation of the different settings can then be performed by visually inspecting the kernel density functions of the corresponding displacement distributions, and population trajectories. The module presents an overview of these trajectories, highlighting which ones would be retained (marked ‘yes’ in green), and which would be removed (marked ‘no’ in red) (see Fig. 9). This allows the user to qualitatively judge if filtering is useful, with the goal of removing artifacts and dead cells. For instance, in the example in Fig. 9, a minimal step threshold of 0.4 μm, together with a minimum of 30% motile steps, removes more than 30% of the tracks for the untreated cells, which indicates over-filtering. A desired level of filtering can be applied for each condition separately, or be set globally for all conditions. Furthermore, a summary is provided for each condition, detailing the number and the percentage of retained cell trajectories, to aid the selection process (see Fig. 10). In the example the threshold was set at 0.1 μm and 30% motility; in this condition the number of retained tracks is only low (~45% retained tracks) in the conditions were high doses of the specific inhibitors (crizotinib, purple curve, lapatinib, olive green curve) were used. This conforms to the fact that these doses were partly toxic in a proliferation test (data not shown).

**Figure 11:**
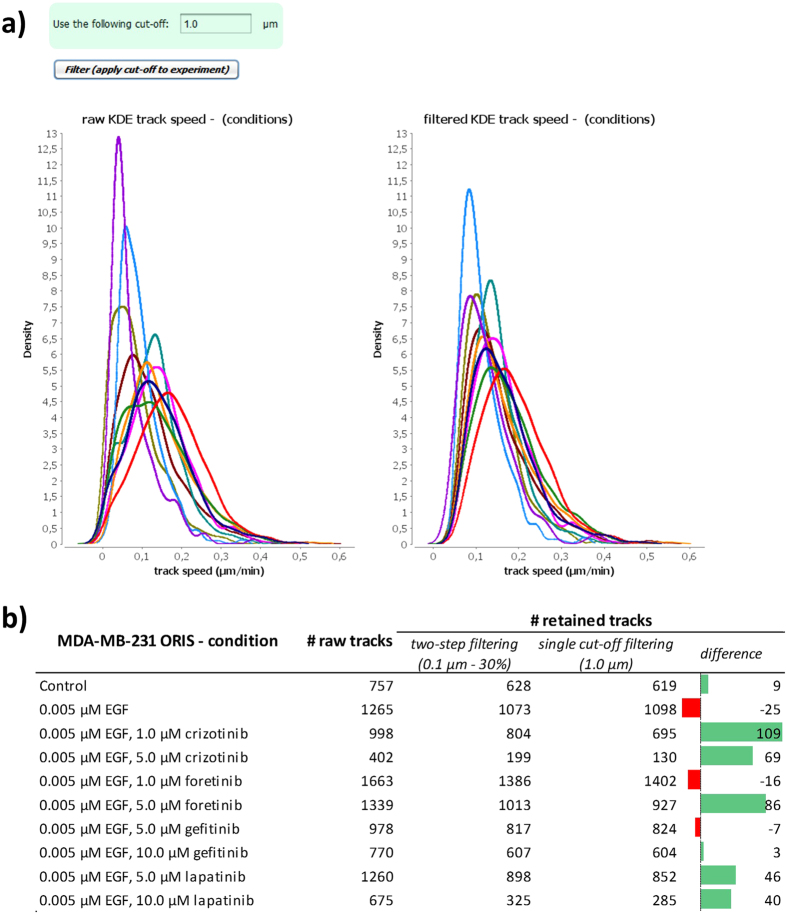
Single cut-off filtering. (**a)** In this option, the user can simply provide a minimal trajectory-centric displacement threshold, and this cut-off is used to filter out cell trajectories whose displacement is below this threshold. This functionality is here shown for all conditions of experiment 2. (**b**) Table reporting the difference in stringency across the two filtering approaches.

This flexible, two-step filtering approach can outperform a single, low cut-off-value for either mean trajectory or cell displacement when it comes to excluding true artifacts. In fact, this two-step filter allows the frequently observed phenomenon of cell pausing during migration to be taken into account (see for example the tracks shown in Fig. 3), and will retain living cells that are only motile in part of the time sequence. Nevertheless, the module also provides the simpler, single-value thresholding option, where all trajectories of cells whose median displacement is below a user-defined threshold are discarded (this single threshold is then applied across all the data sets for the overall experiment). Figure 11a shows this option for the same experiment used in Fig. 10. Figure 11b illustrates the higher stringency when using a single cut-off value for the filtering, given that for several conditions the number of rejected trajectories is higher.

### Data analysis and statistics

**Figure 12:**
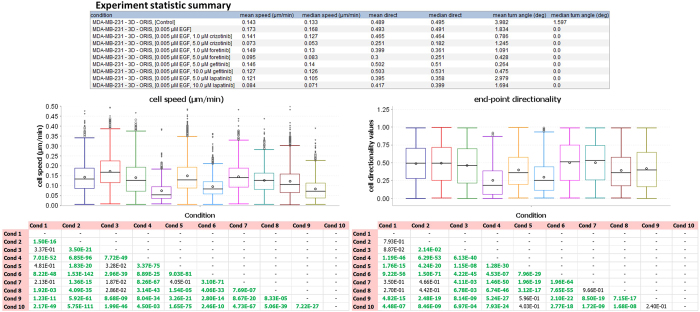
Results views. Parts of final views are shown. The experiment statistics summary is reported in the table. Boxplots are shown for cell speed and end-point directionality values. The two tables at the bottom report p-values from a Mann-Whitney U test performed on speed (left) and directionality (right) distributions. P-values smaller than 0.05 (significance level can be chosen in the tool) are highlighted in green. A multiple hypotheses testing with Benjamini-Hochberg correction is applied. Data shown in this figure are from experiment 2.

Once the data have been controlled for quality, statistical analysis and final interpretation of the experiment can take place. Importantly, at this stage, the user can still choose to analyze the raw data (*i.e.*, without filtering). For each biological condition, statistics on speed, turning angle, and directionality ratio are reported, together with a visualization of the parameter distributions (see Fig. 12).

Subsequently, the user can select a set of conditions on which they wish to perform statistics. Because the migration data generally display a non-parametric distribution and are skewed to higher values, a Mann-Whitney U test is implemented instead of a simpler Student’s t-test. The user can choose the parameter to test (currently either speed *s* or directionality ratio *ep_dr*), and can furthermore opt to correct for multiple hypotheses testing (with either Bonferroni, or Benjamini-Hochberg correction). A table reporting p-values is then shown, and a final data visualization provides a recapitulation of the entire analysis (see Fig. 12). All figures throughout the module can be interactively customized (*e.g.*, axis range, font size of labels) and all results (figures and tables) can be easily copied or exported for downstream reporting.

## Conclusions

The new single-cell analysis module for CellMissy presented here, provides a powerful and largely automated pipeline for high-throughput, single-cell migration experiments downstream of image processing. It supports multi-parametric (speed, directionality), multiscale (step, trajectory, cell population; replicate, condition, multi-sample experiment), and quality-controlled analysis of the differences between tested conditions. It therefore enables more in-depth analyses compared to existing tools (see Table 1). Moreover, given its plugin architecture, it can be further extended and customized in the future to incorporate additional analyses. For example, given the increasing focus on 3D cell migration analysis, more parameters can be added to accommodate for xyz-trajectories. Because this module is integrated in the CellMissy data management system, it is immediately useable and works on all operating systems. Moreover, integration with CellMissy ensures that all quantitative data and associated detailed metadata are stored in a relational database, thus enabling continuous data mining as well as data export and sharing with other CellMissy users.

## Methods

### Sample preparation and image analysis

Ba/F3 cells B-cells expressing chimera of Bcr/Abl were previously described in refs 27 and 28. Ba/F3 and breast carcinoma cells (MDA-MB-231 and BT549) were respectively cultured in RPMI 1640 or DMEM GlutaMAX (Invitrogen) supplemented with 10% heat inactivated fetal bovine serum (FBS, HyClone™). Cells were either left untreated, stimulated with 5 nM epidermal growth factor (EGF, Sigma) or treated with ROCK inhibitor (Tocris), or a receptor tyrosine kinase inhibitor, as indicated. Ba/F3 single cell motility was monitored in 48-well plates; cells (2500 cells/well) were present in 2 mg/ml Matrigel™ (BD Biosciences), and enriched in one layer. MDA-MB-231 and BT549 cells were seeded as a confluent layer on 2 mg/ml collagen around a cell-free zone in a 96-well plate and subsequently overlaid with a second layer of collagen gel (3D cell-exclusion zone assay, Oris™ Platypus, tebu-bio). The experimental conditions are summarized for the Ba/F3 and MDA-MB-231 cells in Table 2. Here, the number of replicates/condition and the number of conditions tested in parallel are reported.

Single cells migrated into the central cell-free zone in time. Migration was monitored using phase-contrast imaging on a Cell^M Live Cell Imaging system (Olympus) every 2.5 or 20 minutes for Ba/F3 and breast cancer cells, respectively. Image processing was performed with a software tool (CELLMIA), developed in collaboration with DCILabs Belgium (http://www.dcilabs.com/). The (x, y) coordinates in time generated by the imaging processing were then automatically imported into CellMissy.

### Implementation

The software here presented is a new module in CellMissy, an open-source and cross-platform package dedicated to the management, storage, and analysis of cell migration data^15^. This new module has been specifically designed to accommodate for single-cell migration data. The “Experiment Manager” of CellMissy provides the means to set up and document an experiment, *i.e.*, specify the experimental details (cell type, assay type, treatment, etc.). Data and metadata (both related to the experimental setup and to the imaging) can be stored into a relational database through a new import module for single-cell migration data.

The new module is written in Java, and inherits the cross-platform support from CellMissy, which is available on any system that supports a Java Virtual Machine version 1.8.0 or above (Windows, Linux, and Mac OS-X). Furthermore, the new module has been built around a plug-in architecture, which means that it can be extended with additional algorithms and functionalities by any interested researchers or developers.

### Code and data availability

The software binary is available at https://github.com/compomics/cellmissy#downloads. Source code is available at https://github.com/compomics/cellmissy, and an archived version is deposited at Figshare [doi: 10.6084/m9.figshare.3421966]. Software is distributed under the Apache2 open source license. Data used for this article are available at the figshare repository [doi: 10.6084/m9.figshare.3413848, link: https://dx.doi.org/10.6084/m9.figshare.3413848).

## Additional Information

**How to cite this article:** Masuzzo, P. *et al*. An end-to-end software solution for the analysis of high-throughput single-cell migration data. *Sci. Rep.* **7**, 42383; doi: 10.1038/srep42383 (2017).

**Publisher's note:** Springer Nature remains neutral with regard to jurisdictional claims in published maps and institutional affiliations.

## Figures and Tables

**Table 1:** Overview of some of the currently available tools/software dedicated to single-cell migration data.

Software	Experimental setup, annotation	Free	Open Source	Cell tracking	Multiple conditions, technical replicates	Quality control	Statistics	Database	Multiscale analysis	Visualization of tracks	Multiparameter visualization	Homepage
**CellMissy single-cell module**	+	+	+	−	+	+	+	+	SC, TC, CP	RP, HMP	RP, HG, AHG, BP, KDP, HMP	https://github.com/compomics/cellmissy
**CellProfiler Tracer**	−	+	+	+	+	+	−	+	TC, CP	RP, HMP	RP, HG, BP, HMP	http://cellprofiler.org/tracer/
**CellTracker**	−	−	+	+	−	−	−	−	CP	RP	RP	http://celltracker.website/about-celltracker.html
**Ibidi Chemotaxis and Migration Tool**	−	+	+	+	−	−	+	−	CP	RP	RP, AHG	http://ibidi.com/xtproducts/en/Software-and-Image-Analysis/Manual-Image-Analysis/Chemotaxis-and-Migration-Tool
**Icy PACT/Icy Track Manager**	−	+	+	+	−	−	−	−	TC, CP	RP	RP, AHG	http://icy.bioimageanalysis.org/protocol/PACT:_a_Pipeline_for_Automated_Cell_Tracking
**ImarisTrack**	−	−	−	+	−	+	+	−	CP	RP	RP, HG	http://www.bitplane.com/imaris/imaristrack
**MetaMorph**	+	−	−	+	+	−	−	−	CP	RP	RP, HG	https://www.moleculardevices.com/systems/metamorph-research-imaging/metamorph-microscopy-automation-and-image-analysis-software
**MotilityLab**	−	+	−	−	−	−	+	−	CP	RP	BP	http://www.motilitylab.net
**TrackMate**	−	+	+	+	−	+	−	−	TC, CP	RP	RP, HG	https://imagej.net/TrackMate
**Volocity**	+	−	−	+	+	−	−	−	CP	RP	RP, HG	http://www.perkinelmer.com/lab-products-and-services/cellular-imaging/tracking-in-volocity.html

**Multiscale analysis** SC: step-centric TC: trajectory-centric CP: cell population **Visualization of tracks/Multiparameter visualization** RP: rose plot HMP: heat-map plate AHG: angular histogram BP: boxplot HG: histogram KDP: kernel density plot.

**Table 2:** Description of the experimental data used in this article to demonstrate the functionalities of the single-cell analysis module in CellMissy.

Experiment number	1	2
**Cell line**	Ba/F3	MDA-MB-231
**Cell type**	B-cell	Mammary adenocarcinoma
**#conditions tested**	6	10
**Conditions**	Bcr-Abl p190^a^, Bcr-Abl p210, or Bcr-Abl p210mut; ± y27632	Control, EGF, crizonib^c^, foretinib^c^, gefitinib^d^, lapatinib^d^
**#replicates/condition**	2	6
**Assay type**	Single cell migration of cells embedded in Matrigel (2 mg/ml), cells enriched in one focal plane	Cell zone exclusion assay^e^ in 3D collagen matrix (2 mg/ml)
**Time-lapse: imaging type, duration, interval, magnification**	Phase-contrast xyt imaging, 6 h, 1.5 min, 20x	Phase-contrast xyt imaging, 48 h, 20 min, 10x
**Image processing: software/output used**	CELLMIA^b^, (x, y) trajectories	CELLMIA^b^, (x, y) trajectories of cells escaping form the confluent sheet

^a^Bcr-Abl oncogene variants (see ref. 27); y27632: Rock inhibitor used at 10 μM.

^b^In house development tool (collaboration Dci-Labs Belgium).

^c^c-Met inhibitors (different concentrations tested).

^d^Epidermal growth factor (EGF)-inhibitors (different concentrations tested).

^e^see ref. 29.

## References

[b1] Luster A. D., Alon R. & von Andrian U. H. Immune cell migration in inflammation: present and future therapeutic targets. Nat. Immunol. 6, 1182–1190 (2005). 16369557 10.1038/ni1275

[b2] Friedl P. & Gilmour D. Collective cell migration in morphogenesis, regeneration and cancer. Nature reviews. Molecular cell biology 10, 445–457 (2009). 19546857 10.1038/nrm2720

[b3] Aman A. & Piotrowski T. Cell migration during morphogenesis. Dev. Biol. 341, 20–33 (2010). 19914236 10.1016/j.ydbio.2009.11.014

[b4] Slaney C. Y., Kershaw M. H. & Darcy P. K. Trafficking of T cells into tumors. Cancer Res. 74, 7168–7174 (2014). 25477332 10.1158/0008-5472.CAN-14-2458

[b5] Goichberg P. Current Understanding of the Pathways Involved in Adult Stem and Progenitor Cell Migration for Tissue Homeostasis and Repair. Stem Cell Rev, doi: 10.1007/s12015-016-9663-7 (2016). 27209167

[b6] Sanz-Moreno V. & Marshall C. J. The plasticity of cytoskeletal dynamics underlying neoplastic cell migration. Current opinion in cell biology 22, 690–6 (2010). 20829016 10.1016/j.ceb.2010.08.020

[b7] Nürnberg A., Kitzing T. & Grosse R. Nucleating actin for invasion. Nat Rev Cancer 11, 177–187 (2011). 21326322 10.1038/nrc3003

[b8] Doyle A. D., Petrie R. J., Kutys M. L. & Yamada K. M. Dimensions in cell migration. Curr. Opin. Cell Biol. 25, 642–649 (2013). 23850350 10.1016/j.ceb.2013.06.004 PMC3758466

[b9] Kumar K. S. . Computer-assisted quantification of motile and invasive capabilities of cancer cells. Sci Rep 5, 15338 (2015). 26486848 10.1038/srep15338 PMC4614254

[b10] Decaestecker C., Debeir O., Van Ham P. & Kiss R. Can anti-migratory drugs be screened *in vitro*? A review of 2D and 3D assays for the quantitative analysis of cell migration. Medicinal research reviews 27, 149–176 (2007). 16888756 10.1002/med.20078

[b11] Kramer N. . *In vitro* cell migration and invasion assays. Mutation research 752, 10–24 (2013). 22940039 10.1016/j.mrrev.2012.08.001

[b12] De Wever O. . Single cell and spheroid collagen type I invasion assay. Methods in molecular biology (Clifton, N.J.) 1070, 13–35 (2014). 10.1007/978-1-4614-8244-4_2 24092429

[b13] Masuzzo P., Van Troys M., Ampe C. & Martens L. Taking Aim at Moving Targets in Computational Cell Migration. Trends Cell Biol, doi: 10.1016/j.tcb.2015.09.003 (2015). 26481052

[b14] Gebäck T., Schulz M. M. P., Koumoutsakos P. & Detmar M. TScratch: a novel and simple software tool for automated analysis of monolayer wound healing assays. BioTechniques 46, 265–74 (2009). 19450233 10.2144/000113083

[b15] Masuzzo P. . CellMissy: a tool for management, storage and analysis of cell migration data produced in wound healing-like assays. Bioinformatics 29, 2661–2663 (2013). 23918247 10.1093/bioinformatics/btt437 PMC3789541

[b16] Jaqaman K. . Robust single-particle tracking in live-cell time-lapse sequences. 5 (2008). 10.1038/nmeth.1237 PMC2747604 18641657

[b17] Bray M.-A. & Carpenter A. E. CellProfiler Tracer: exploring and validating high-throughput, time-lapse microscopy image data. BMC Bioinformatics 16, 368 (2015). 10.1186/s12859-015-0759-x PMC4634901 26537300

[b18] Sebag A. S. . A generic methodological framework for studying single cell motility in high-throughput time-lapse data. Bioinformatics 31, i320–i328 (2015). 26072499 10.1093/bioinformatics/btv225 PMC4765885

[b19] Chenouard N. . Objective comparison of particle tracking methods. Nat Meth 11, 281–289 (2014). 10.1038/nmeth.2808 PMC4131736 24441936

[b20] Mokhtari Z. . Automated Characterization and Parameter-Free Classification of Cell Tracks Based on Local Migration Behavior. PLoS ONE 8, e80808 (2013). 24324630 10.1371/journal.pone.0080808 PMC3855794

[b21] Somaweera H., Ibraguimov A. & Pappas D. A review of chemical gradient systems for cell analysis. Anal. Chim. Acta 907, 7–17 (2016). 26802998 10.1016/j.aca.2015.12.008

[b22] Stadler M. . Increased complexity in carcinomas: Analyzing and modeling the interaction of human cancer cells with their microenvironment. Seminars in Cancer Biology 35, 107–124 (2015). 26320002 10.1016/j.semcancer.2015.08.007

[b23] Birmingham A. . Statistical Methods for Analysis of High-Throughput RNA Interference Screens. Nat Methods 6, 569–575 (2009). 19644458 10.1038/nmeth.1351 PMC2789971

[b24] Perlman Z. E. . Multidimensional drug profiling by automated microscopy. Science 306, 1194–1198 (2004). 15539606 10.1126/science.1100709

[b25] Singh D. K. . Patterns of basal signaling heterogeneity can distinguish cellular populations with different drug sensitivities. Mol. Syst. Biol. 6, 369 (2010). 20461076 10.1038/msb.2010.22 PMC2890326

[b26] Beltman J. B., Marée A. F. M. & de Boer R. J. Analysing immune cell migration. Nat Rev Immunol 9, 789–798 (2009). 19834485 10.1038/nri2638

[b27] Rochelle T. . p210bcr-abl induces amoeboid motility by recruiting ADF/destrin through RhoA/ROCK1. FASEB J 27, 123–134 (2013). 23047898 10.1096/fj.12-205112

[b28] Daubon T. . Differential motility of p190bcr-abl- and p210bcr-abl-expressing cells: respective roles of Vav and Bcr-Abl GEFs. Oncogene 27, 2673–2685 (2008). 18059343 10.1038/sj.onc.1210933

[b29] Hulkower K. I. & Herber R. L. Cell migration and invasion assays as tools for drug discovery. Pharmaceutics 3, 107–24 (2011). 24310428 10.3390/pharmaceutics3010107 PMC3857040

